# Assessing kidney stone composition using smartphone microscopy and deep neural networks

**DOI:** 10.1002/bco2.137

**Published:** 2022-01-06

**Authors:** Ege Gungor Onal, Hakan Tekgul

**Affiliations:** ^1^ Department of Bioengineering University of Illinois at Urbana‐Champaign Champaign Illinois USA; ^2^ Department of Computer Engineering Georgia Institute of Technology Atlanta Georgia USA

**Keywords:** artificial intelligence, convolutional neural network, kidney stone, machine learning, point‐of‐care testing, smartphone microscopy, urolithiasis, urology

## Abstract

**Objectives:**

To propose a point‐of‐care image recognition system for kidney stone composition classification using smartphone microscopy and deep convolutional neural networks.

**Materials and methods:**

A total of 37 surgically extracted human kidney stones consisting of calcium oxalate (CaOx), cystine, uric acid (UA) and struvite stones were included in the study. All of the stones were fragmented from percutaneous nephrolithotomy (PCNL). The stones were classified using Fourier transform infrared spectroscopy (FTIR) analysis before obtaining smartphone microscope images. The size of the stones ranged from 5 to 10 mm in diameter. Nurugo 400× smartphone microscope (Nurugo, Seoul, Republic of Korea) was functionalized to acquire microscopic images (magnification = 25×) of dry kidney stones using iPhone 6s+ (Apple, Cupertino, CA, USA). Each kidney stone was imaged in six different locations. In total, 222 images were captured from 37 stones. A novel convolutional neural network architecture was built for classification, and the model was assessed using accuracy, positive predictive value, sensitivity and F1 scores.

**Results:**

We achieved an overall and weighted accuracy of 88% and 87%, respectively, with an average F1 score of 0.84. The positive predictive value, sensitivity and F1 score for each stone type were respectively reported as follows: CaOx (0.82, 0.83, 0.82), cystine (0.80, 0.88, 0.84), UA (0.92, 0.77, 0.85) and struvite (0.86, 0.84, 0.85).

**Conclusion:**

We demonstrate a rapid and accurate point of care diagnostics method for classifying the four types of kidney stones. In the future, diagnostic tools that combine smartphone microscopy with artificial intelligence (AI) can provide accessible health care that can support physicians in their decision‐making process.

## 1 INTRODUCTION

Kidney stones (calculi) are mineral deposits of crystalline and organic components formed when urine is supersaturated with minerals and/or organic components.^1^ The formation of kidney stones (nephrolithiasis) is a common condition. According to the most recent National Health and Nutrition Evaluation Survey, the prevalence of self‐reported kidney stones between 2013 and 2014 was 10.1%; the weighted prevalence of kidney stones was 10.9% (9.1–12.6) for males and 9.4% (7.6–11.1) for females.^2^ There are several underlying risk factors for the formation of kidney stones; family history, race/ethnicity, systemic disorders, environmental factors, dietary factors and urinary factors seem to all play a role in the development of kidney stones.^3^ The abundance of each type of kidney stone varies with type: calcium stones comprise around 60–80% of all kidney stones, uric acid 8–10%, struvite 7–8%, and cystine 0.1–1%.^1^, ^3^

Kidney stones can be treated with minimally invasive surgical techniques such as Extracorporeal shock wave lithotripsy, ureteroscopic lithotripsy, and percutaneous nephrolithotomy (PCNL).^4^ Following the kidney stone extraction and stone analysis, clinicians determine stone composition to prescribe treatment or diet for preventative measures.^5^ The recurrence rate is approximately 50% in 5–10 years and 75% in 20 years without preventive treatment.^6^ Hence, understanding and detecting the formation of specific types of kidney stone is crucial for prescribing treatment to prevent recurrence.

The gold standard for kidney stone analysis in physical analytic methods are Fourier transform infrared spectroscopy (FTIR) and X‐ray diffraction.^7^ Both methods are widely used and considered to be very accurate and reliable for kidney stone analysis. However, there are certain limitations; both FTIR and X‐ray diffraction need trained laboratory personnel and specialized equipment in a laboratory setting. Due to these limitations, clinicians need to send kidney stones to specific testing centres, and this practice is both time consuming and costly for each analysis. Therefore, there is a need for a rapid and accurate point‐of‐care device for kidney stone analysis in daily clinical practice.

Over the past couple of decades, artificial intelligence (AI) has become a significant research area in medical diagnostics and analytics.^8^ There has been ongoing research in building image‐based diagnosis systems for many medical specialties.^9^ Similarly, the adoption of AI in urology is a growing field of interest.^10^, ^11^ Recently, Black et al. reported kidney stone classification using deep learning and digital camera images.^12^

The aim of the present study was to propose an image recognition system that can accurately detect the type of kidney stones using a data set of smartphone‐based microscopic images. To our knowledge, this is the first study for combining smartphone microscopy with deep learning to classify kidney stone types.

## 2 MATERIALS AND METHODS

**FIGURE 1:**
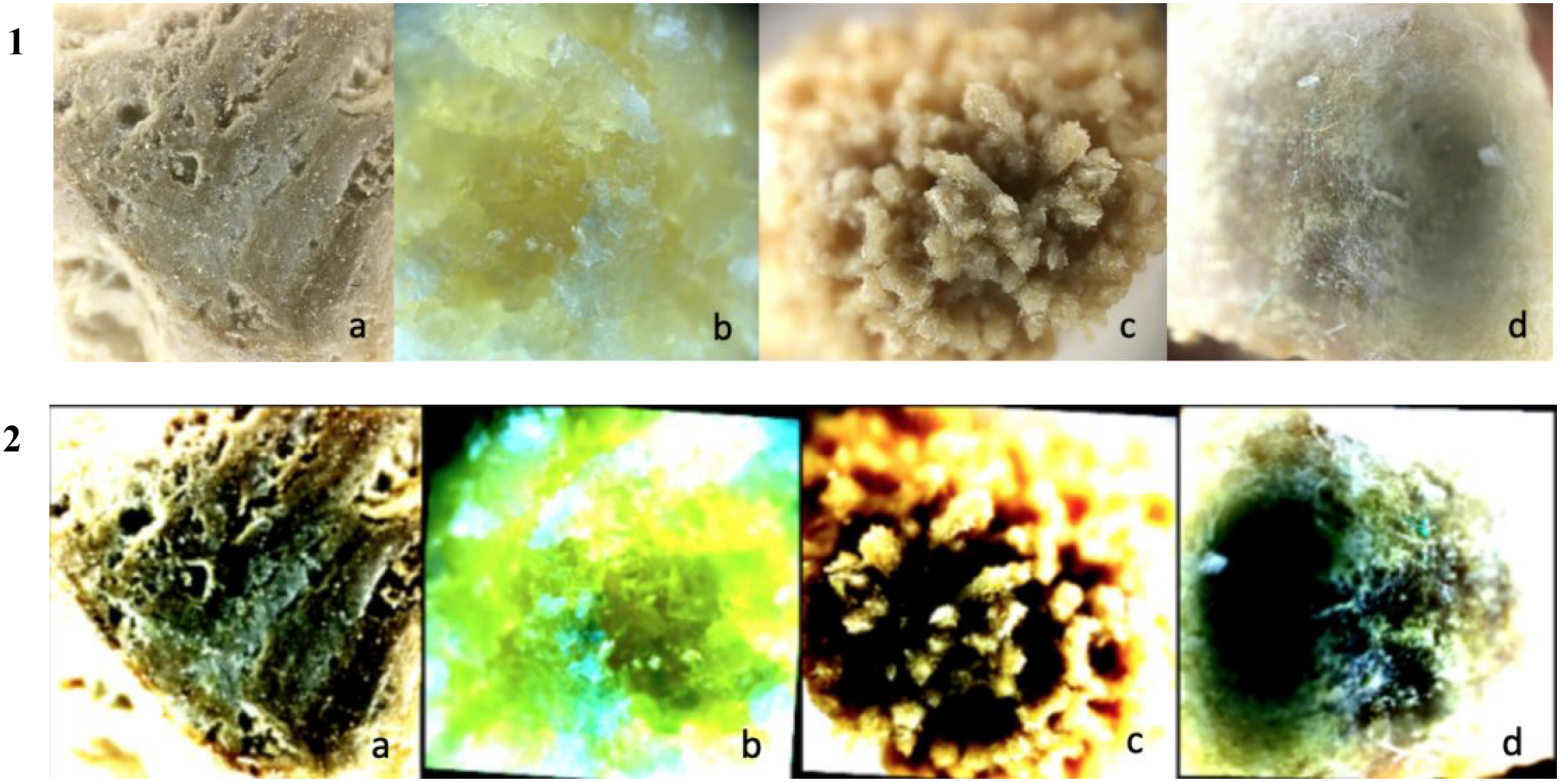
Microscopic images of different types of kidney stones before (1) and after (2) image pre‐processing. Struvite (a), cystine (b), calcium oxalate (c), uric acid (d). (magnification = 25×)

A total of 37 surgically extracted human kidney stones consisting of calcium oxalate (CaOx) (7 calcium CaOx‐monohydrate, 7 CaOx‐dihydrate, 6 CaOx‐monohydrate + CaOx‐dihydrate) (*n* = 20, CaOx), cystine (*n* = 10), uric acid (*n* = 4, UA), and struvite stones (*n* = 3) were included in the study. All of the stones were fragmented from PCNL. The stones were classified using FTIR analysis before obtaining smartphone microscope images. The size of the stones ranged from 5 to 10 mm in diameter. The stones were obtained between 2018 and 2020 from Bulent Onal MD, Istanbul University‐Cerrahpasa, Cerrahpasa School of Medicine, Istanbul, Turkey. All of the stones were preserved in a dry state and as extracted from the patients. Nurugo 400× smartphone microscope (Nurugo, Seoul, Republic of Korea) was functionalized to acquire microscopic images (magnification = 25×) of dry kidney stones using iPhone 6s+ (Apple, Cupertino, CA, USA). The smartphone‐microscope was hand‐held to obtain images of the kidney stones. Distance from objective and focus were manually adjusted by the user to mirror real‐life point‐of‐care usage conditions. Each kidney stone was imaged in six different locations. In total, 222 images were captured from 37 stones. A summary of our data set for different stone types is presented in Table 1. Microscopic image samples of different types of kidney stones are shown in Figure 1.

**TABLE 1:** Number of kidney stones and the total number of images for each stone type

Stone types	No. of stones	Total no. images
Cystine	10	60
Calcium oxalate	20	120
Struvite	3	18
Uric acid	4	24

**FIGURE 2:**
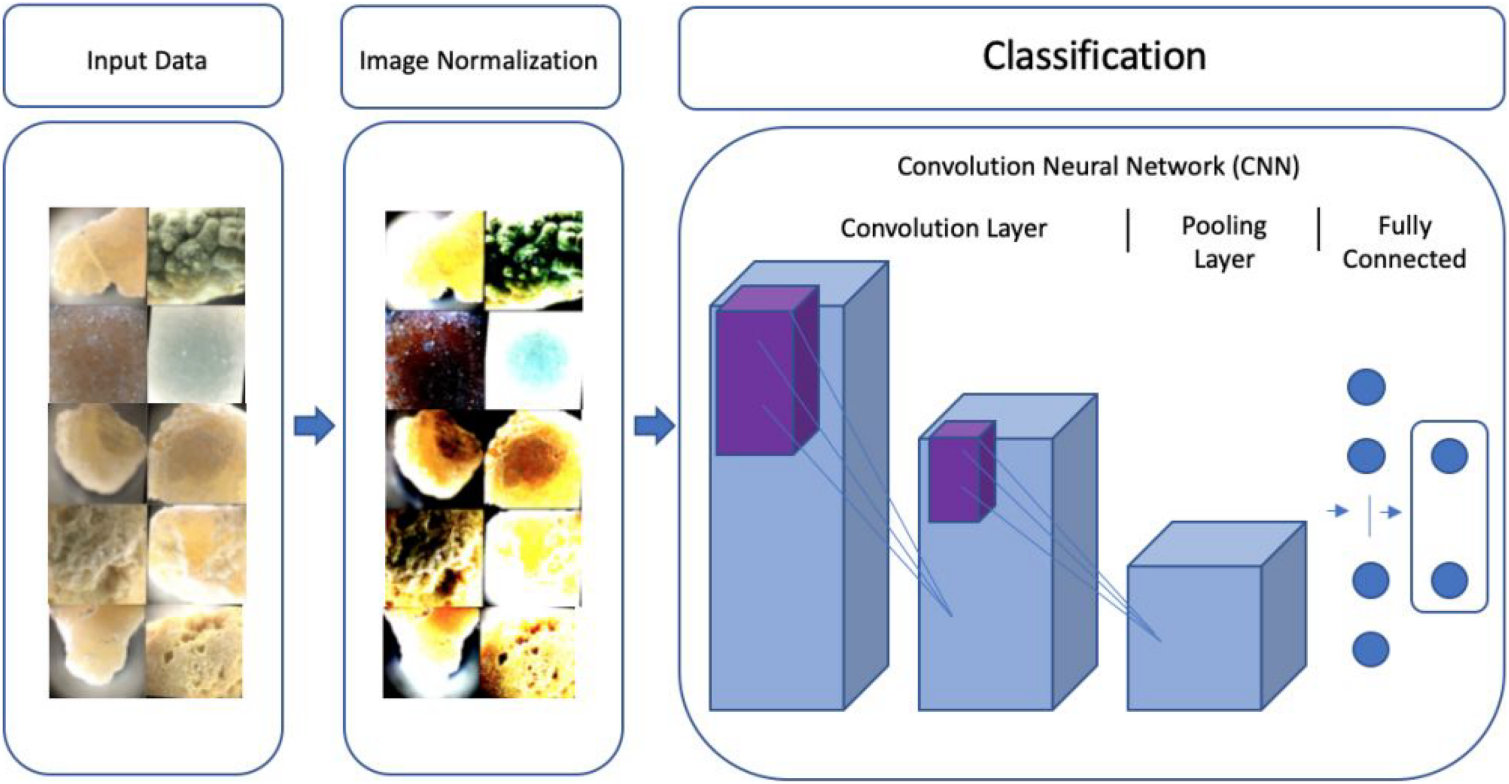
Proposed machine learning pipeline for kidney stone classification

We propose a machine learning pipeline that can be used for kidney stone classification. Our approach consisted of multiple stages, starting with data acquisition and ending with outputting the correct kidney stone type from our classifier. Each stage output in our approach was provided as input to the next stage. Our pipeline and approach for this kidney stone problem was summarized in Figure 2.

Upon the creation of our data set for microscopic images, we utilized image pre‐processing for classification. Each microscopic image was resized into 224 × 224 pixels by cropping at the centre. Then, random horizontal split and random 5° rotation was applied to random sets of images to increase generalization. Finally, each image was normalized with specific mean and standard deviation values for each RGB channel. These specific values were selected after many years of image processing research for convolutional neural networks (CNNs).^13^ Table 2 presents the image processing parameters in detail. For all of our image processing operations, we used PyTorch's^13^ transforms package. Some sample images after image pre‐processing are presented in Figure 1. After the images are processed, the data set is split into training and testing sets with a ratio of 70% and 30%, respectively. Thus, it is important to note that 26 stones' images are used for training, and 11 stones' images are used for testing.

**TABLE 2:** Image pre‐processing parameters for PyTorch

Image size	224 × 224 pixels
Random rotation	5 degrees
Random horizontal flip probability	20%
Mean normalization constants (R,G,B)	(0.485, 0.456, 0.406)
STD normalization constants (R,G,B)	(0.229, 0.224, 0.225)

**FIGURE 3:**
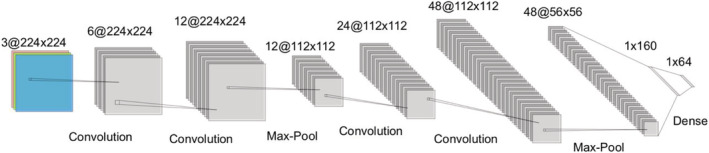
Proposed neural network architecture

Even though most related works for similar classification problems in medical imaging use the ResNet50 or GoogleNet architecture, the complex structure and unnecessary number of layers hinder the classification's efficiency. Training such complex architectures might take days with large data sets. Therefore, we developed our own novel CNN architecture. The architecture has four convolutional layers, two pooling layers and two fully connected layers. The proposed architecture is shown in Figure 3. Python 3.6 and PyTorch^13^ was used for image processing and CNN algorithms. Our model was trained using a 2.3‐GHz Intel Core i5 CPU with 8‐GB RAM. All the hyperparameter values were presented in Table 3. The training of our proposed model took an average of 45 min. After training, the machine learning model can classify between the four types of kidney stones in less than 30 s.

**TABLE 3:** Parameters used for the convolutional neural network (CNN)

Loss function	Cross‐entropy
Learning rate	0.001
Weight decay	1.00E‐05
Optimizer	Adam
Number of epochs	60
Dropout rate	0.3

The overall and weighted accuracy, positive predictive value, sensitivity, F1 scores, and confusion matrix for the classification model were recorded. Accuracy represents the number of correct predictions divided by the total number of predictions. Because our study has an imbalanced data set, the classifier might be biased towards the type of kidney stone that occurs the most in our data set. Therefore, there is a need to analyse other metrics, such as weighted accuracy, positive predictive value, sensitivity and F1 scores. Weighted accuracy is computed by taking the mean of the rate of correct predictions in every class. Sensitivity is computed by the ratio of true positives to the sum of true positives and false negatives. On the other hand, positive predictive value is computed by the ratio of true positives to the sum of true positives and false positives. F1 score is the harmonic mean of positive predictive value and sensitivity which expresses the performance of each class in our classifier. Confusion matrices are used to explain the performance of classifiers for each class. The actual versus predicted values represent unseen images (30%) (*n* = 11) used in testing by our classifier per stone type.

## 3 RESULTS

**FIGURE 4:**
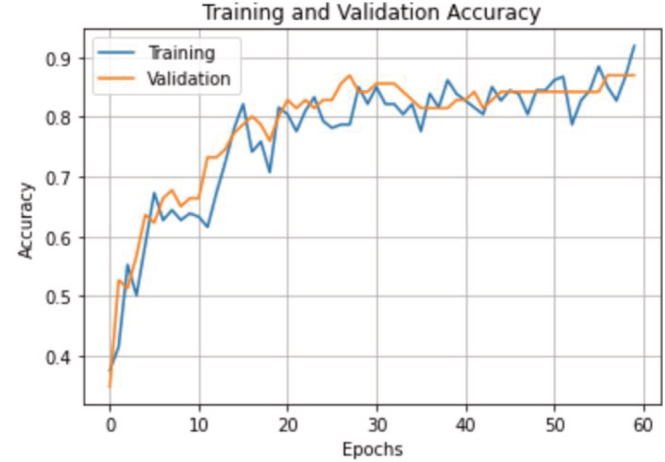
Training and validation accuracy results with respect to number of epochs

We achieved an overall and weighted accuracy of 88% and 87%, respectively, with an average F1 score of 0.84. Training and validation accuracies for the number of epochs are shown in Figure 4. One epoch corresponds to all training images processed once.

The positive predictive value, sensitivity and F1 score for each stone type were respectively reported in Table 4 as follows: CaOx (0.82, 0.83, 0.82), cystine (0.80, 0.88, 0.84), UA (0.92, 0.77, 0.85) and struvite (0.86, 0.84, 0.85).

**TABLE 4:** Positive predictive value, sensitivity and F1 score for each kidney stone types

Stone types	Positive predictive value	Sensitivity	F1 score
CaOx	0.82	0.83	0.82
Cystine	0.80	0.88	0.84
Struvite	0.86	0.84	0.85
Uric acid	0.92	0.77	0.85

**FIGURE 5:**
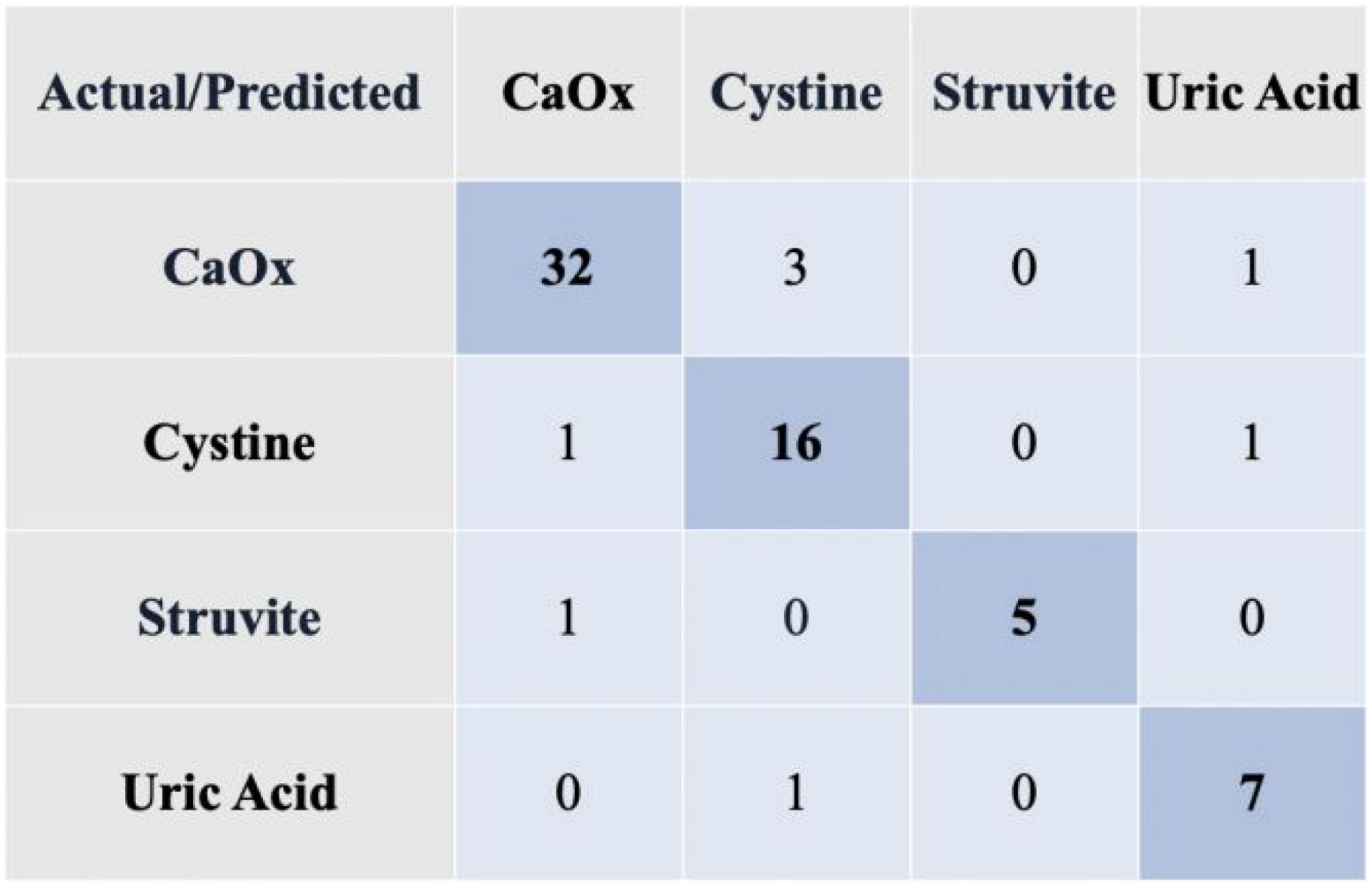
Confusion matrix of our kidney stone classifier (30% of the total images from each stone type were used as unseen images for testing)

The confusion matrix in Figure 5 summarizes the predictions made by our classifier, where each column represents the predicted stone type, and each row shows the actual stone type.

## 4 DISCUSSION

This study demonstrates a bench‐to‐bedside platform for rapid classification of kidney stones using smartphone microscopic images. Our proposed deep learning pipeline includes image processing, data augmentation, and a novel CNN architecture. After training our model with 154 images and testing on 68 unseen kidney stone images, we achieved an accuracy of 88% with an average F1 score of 0.84. To our knowledge, this is the first study for combining smartphone microscopy with deep learning to classify kidney stone types. Our methodology is rapid and easy to use by medical practitioners and urologists.

In two previous studies, Serrat et al. and Black et al. have demonstrated digital image‐based computational methods to determine kidney stone composition.^11^, ^12^ Serrat et al. used a data set of 3632 images from 454 kidney stones, including eight kidney stone types (calcium oxalate monohydrate, calcium oxalate dihydrate, mixed calcium oxalate and hydroxyapatite, hydroxyapatite, struvite, brushite, uric acid anhydrous and dihydrate, mixed uric acid and calcium oxalate). They captured a total of four images per kidney stone using a conventional camera. Each sample stone was divided into two fragments; each fragment's external and internal sides were imaged using visible lighting. A traditional machine‐learning approach (random forest classifier) was used to determine kidney stone types based on manually obtained features such as local binary patterns, colour histogram and grey level histogram obtained from images. Lastly, the authors included urinary pH as an additive feature to improve classification. They achieved an overall accuracy of 63%. Although Serrat et al. used a larger data set, the overall accuracy for classification (63%) was lower than our study (88%). We believe this is due to three main reasons: types of kidney stones used, image collecting methodology, and computational approaches for classification. In our study, we accepted CaOx‐monohydrate, CaOx‐dihydrate, and their mixed composition as CaOx stones. Hence, our study includes four types of kidney stones: calcium oxalate, uric acid, cystine and struvite. On the other hand, Serrat et al. analysed subclasses of CaOx separately and further included additional mixed stone types in their study. We functionalized smartphone microscopy for image collecting methodology to analyse magnified crystalline structures of kidney stones, whereas Serrat et al. used digital images of kidney stones without magnification. Serrat et al. used internal and external images; similarly, because the stones were previously fragmented with PCNL, we were able to capture both internal and external surfaces of stones through imaging in six different locations. The computational approach used by Serrat et al. consisted of manually extracting image features and feeding them into a random forest classifier to determine kidney stone types. We used CNNs to eliminate any bias that can be introduced by using manual feature extraction. CNN autonomously extracts all useful features for classification, eliminating bias, and yielding higher accuracy.

Similar to our study, Black et al. reported using CNNs to classify kidney stones.^12^ Their study included 127 images of 63 kidney stones, including uric acid, calcium oxalate monohydrate, struvite, cystine and brushite stones. They used a total of two images per kidney stone, capturing the surface and inner core using a digital camera. Black et al. reported an overall accuracy of 85%. Our kidney stone classifier accuracy (88%) was similar to the one reported in Black et al. (85%). This may be explained by similar data sets and kidney stone types used. However, in our study, we used smartphone microscopy to obtain magnified images of different parts of each kidney stone, whereas Black et al. captured digital images without magnification. Black et al. used internal and external images; as we outlined earlier, we also captured both internal and external sites through imaging in six different locations because the stones were previously fragmented with PCNL. Using this methodology, we obtained a larger set of images (222) than Black et al. (127). In addition, the image classification method used by Black et al. is a widely used conventional CNN architecture (ResNet, Microsoft), whereas we introduced a novel CNN architecture specifically built for classifying kidney stone types. Lastly, we used a train‐test split approach for testing our classifier and tested on 30% of the unseen kidney stones images, unlike the leave one out approach from Black et al.

Last, we believe that our technique has the potential to use AI for linking stone observations with patient data. In most cases such data will be recent data, acquired from the patient at the time when the stone problem presented or a few years before that in the case of recurrence. In that respect, the characteristics of the external parts of a stone will be linked to the current situation of the patient while the origin of the stone may be decades older and related to events that occurred many years earlier in a time period of during which probably no data are available for that patient.^14^

Our current study has certain limitations. The low natural frequency of occurrence of some types of kidney stones was an obstacle in collecting data for representing all types of kidney stones in our data set. Our data set only represents around 90% of kidney stone cases, and the current data set has imbalances in the number of stones for each respective type. In the future, we want to include other kidney stone types such as brushite and mixed stones to expand the reach of our classification and improve our data imbalance. The collection of a larger image data will also improve CNN accuracy. Although our current study has limitations, it serves as a good first step towards demonstrating an automated kidney stone classification method using smartphones.

## CONCLUSIONS

We demonstrate a rapid and accurate point of care diagnostics method for classifying the four main types of kidney stones. Our work demonstrates the significance of smartphone microscopy and deep learning for future medical diagnostics platforms. In the future, diagnostic tools that combine smartphone microscopy with AI can provide accessible health care that can support physicians in their decision‐making process.

## DISCLOSURE OF INTERESTS

None.

## AUTHOR CONTRIBUTIONS

Conception and design of the study: Ege Gungor Onal; Acquisition of data: Ege Gungor Onal; Computational analysis: Hakan Tekgul; Drafting the article: Ege Gungor Onal and Hakan Tekgul; Revision the article: Ege Gungor Onal and Hakan Tekgul; Final approval of the article before the submission: Ege Gungor Onal and Hakan Tekgul; Contributed to the study conception and design: Ege Gungor Onal and Hakan Tekgul.
